# An evolutionarily-unique heterodimeric voltage-gated cation channel found in aphids

**DOI:** 10.1016/j.febslet.2015.01.020

**Published:** 2015-02-27

**Authors:** Joanna S. Amey, Andrias O. O’Reilly, Mark J. Burton, Alin M. Puinean, Ian R. Mellor, Ian R. Duce, Linda M. Field, B.A. Wallace, Martin S. Williamson, T.G. Emyr Davies

**Affiliations:** a Department of Biological Chemistry and Crop Protection, Rothamsted Research, Harpenden, Hertfordshire, United Kingdom; b Institute of Structural and Molecular Biology, Birkbeck College, University of London, London, United Kingdom; c School of Life Sciences, Faculty of Medicine and Health Sciences, University of Nottingham, United Kingdom; d School of Natural Sciences and Psychology, Liverpool John Moores University, Liverpool, United Kingdom; e Department of Cell Physiology and Pharmacology, College of Medicine, Biological Sciences and Psychology, University of Leicester, United Kingdom

**Keywords:** BLAST, basic local alignment search tool, bp, base pair, cDNA, DNA complementary to RNA, DDT, dichlorodiphenyltrichloroethane, kb, kilobase(s) or 1000 base pairs, MYA, million years ago, Na_v_, voltage-gated sodium channel, NCBI, The National Center for Biotechnology Information, PCR, polymerase chain reaction, RACE, Rapid Amplification of cDNA ends, RNA, ribonucleic acid, TM, trans membrane, TTX, tetrodotoxin, Voltage-gated sodium channel, Aphid, Evolution, Tetrodotoxin

## Abstract

We describe the identification in aphids of a unique heterodimeric voltage-gated sodium channel which has an atypical ion selectivity filter and, unusually for insect channels, is highly insensitive to tetrodotoxin. We demonstrate that this channel has most likely arisen by adaptation (gene fission or duplication) of an invertebrate ancestral mono(hetero)meric channel. This is the only identifiable voltage-gated sodium channel homologue in the aphid genome(s), and the channel’s novel selectivity filter motif (DENS instead of the usual DEKA found in other eukaryotes) may result in a loss of sodium selectivity, as indicated experimentally in mutagenised Drosophila channels.

## Highlights

• Aphids have a unique heterodimeric voltage-gated sodium channel.
• The aphid channel has an atypical ion-selectivity filter (DENS rather than DEKA).
• The channel’s novel selectivity filter may result in a loss of sodium selectivity.
• This is the only identifiable voltage-gated sodium channel in aphid genome(s).
• This channel has most likely arisen by gene fission or gene duplication.

## 1 Introduction

**Fig. 1:**
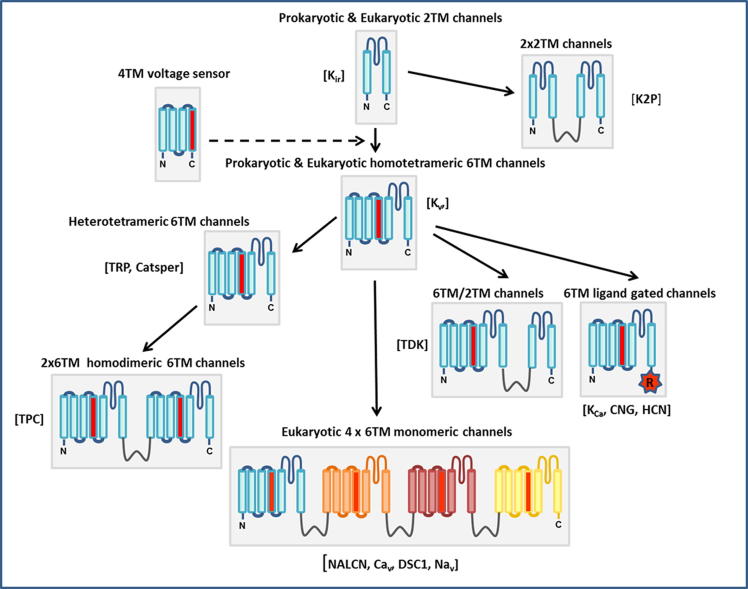
Scheme of the proposed evolutionary relationships between members of the voltage-gated ion channel family. The inwardly rectifying K^+^ channels (*K*_ir_) represent the simplest structural motif in the ion channel superfamily in eukaryotes; their 2TM structure is highly representative of the pore domain found in many ancestral prokaryotic and eukaryotic channels. In the majority of eukaryotic channels, the 2TM motif has been augmented by 4 additional TM segments comprising a voltage-sensing domain (similar to that in the proton channel H_v_1). Thus K_v_ channels (and prokaryotic Na_v_s) are homo-tetrameric assemblies of 6TM subunits. It is currently thought that the single-domain 6TM K_v_ channels underwent two rounds of internal gene duplication leading to multi-domain Na_v_s and Ca_v_s, which resulted in superior kinetics and modulation of channel activation, inactivation, and recovery from inactivation. Two-repeat 6TM homodimeric channels, which may be an evolutionary intermediate, exist in the two-pore channel (TPC) family of Ca^2+^-permeable channels [51].

Voltage-gated sodium channels (Na_v_s) mediate the rising phase of the action potential in the majority of innervated metazoans. Due to this critical role in neurotransmission the channels are invariably highly conserved across phyla. Eukaryotic Na_v_s are multi-domain proteins, consisting of four non-identical domains (DI-DIV), with each domain comprising six transmembrane (TM) segments (S1–S6) containing a voltage sensor (S1–S4) and a membrane-spanning pore region (S5–S6). Typically, their ion (Na^+^) selectivity filter consists of one loop from each domain (located between S5 and S6 of the pore region) which collectively form a ‘DEKA’ (DI-aspartate; DII-glutamate; DIII-lysine; DIV-alanine) amino acid sequence motif at the entry to the pore. In evolution terms, Na_v_s are considered to be the most recent members of a large family of ion channels that includes voltage- and ligand-gated K^+^ channels, Ca^2+^ channels and several non-selective channels [1]. Prokaryotic channels, which are the likely progenitors, consist of single domain polypeptides that self-assemble to form functional tetrameric channels. These have some striking similarities to vertebrate monomeric four domain (4x6TM) Na_v_ and Ca_v_ channels, so it is likely that the multi-domain channels arose by multiple cycles of gene duplication and fusion from an ancestral single-domain protein [2] (Fig. 1). Contemporary Na_v_ channels are thought to have evolved from a family of channels called Na_v_2 [3] which, because of their unique pore sequence (DKEA or DEEA), preferentially conduct Ca^2+^ [4]. Although this family of cation channels has apparently been lost in vertebrates, they can still be found in invertebrates [5].

Higher order metazoans typically contain multiple genes encoding 4x6TM Na_v_ channels with different characteristics and functions [6]. These isoforms can be differentiated pharmacologically based on their sensitivity to the pore blocking toxin, tetrodotoxin (TTX). In contrast, all insects thus far studied have only one 4x6TM Na_v_ gene, with alternative splicing of exons imparting functional variability [7] and all channel variants exhibiting high sensitivity to TTX. The 4x6TM Na_v_s also characteristically bind modulatory drugs and other neurotoxins including local anaesthetics, voltage-sensor disabling scorpion toxins and insecticides such as DDT and the synthetic pyrethroids [8].

It was unexpected when genome annotation predictions for the pea aphid, *Acyrthosiphon pisum* (Harris) [9] identified two genes encoding putative ApNa_v_1 sequences, correlating with LOC100158802 (NCBI accession XP_008183364.1) encoding DI and DII and LOC100164620 (accession XP_001949648.2) encoding DIII and DIV. This suggested that the pea aphid has a two subunit channel. In this study, we further analysed this genomic data and sought corroborating evidence for the existence of a two subunit channel in aphids that reprises the role of a multi-domain Na_v_1 as found in other insect species. This led us to the identification of an evolutionarily-unique heterodimeric voltage-gated cation channel in aphids.

## 2 Results

**Supplementary Fig. 1:**
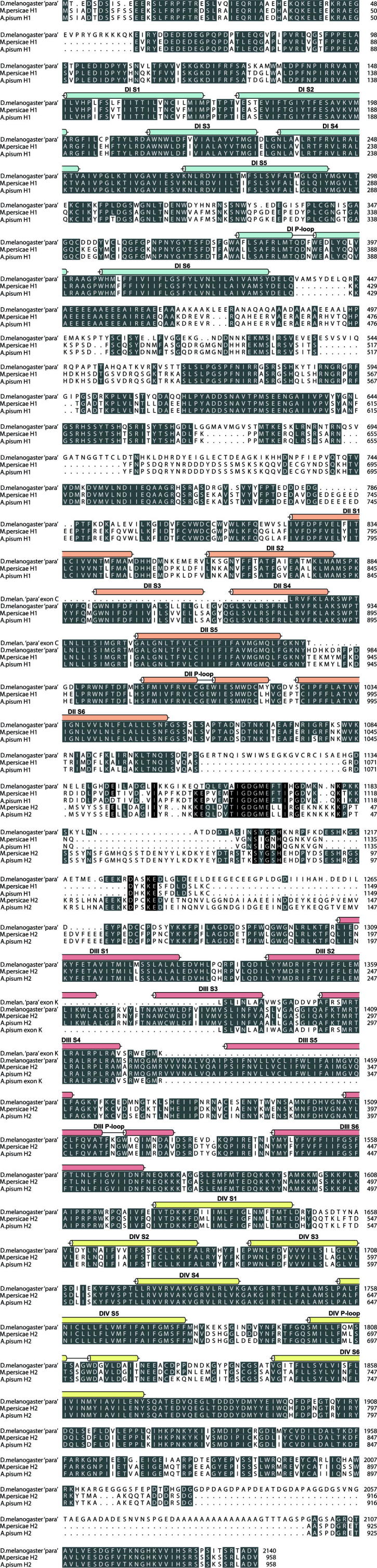
Alignment of fruitfly (*D. melanogaster*) DmNa_v_1 (para) voltage-gated sodium channel and the pea aphid (*A. pisum*) and peach potato aphid (*M. persicae*) channel H1 and H2 subunits. The *M. persicae* cDNAs are highly conserved at the amino acid level with the *A. pisum* H1 and H2 sequences (98.7% (DI–DII); 97.7% (DIII–DIV)). Transmembrane helices from different domains are indicated by bars in different colours above the sequences. Identical amino acids are highlighted in grey. [Note the apparent evolutionary sequence duplication (highlighted in black) in the DII-DIII intracellular loop to generate the novel 5′ end for H2].

**Fig. 2:**
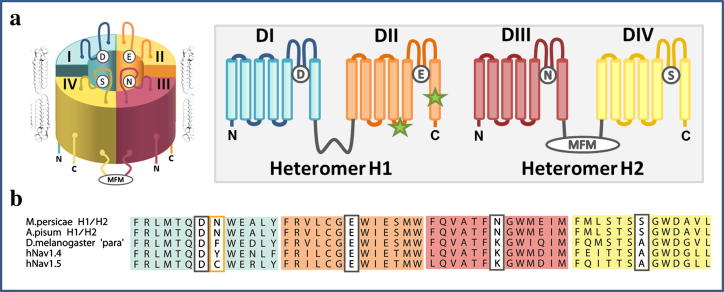
(a) Models for the H1 and H2 subunits. Each consists of two 6TM domains, with the approximate position of the fast inactivation particle ‘MFM’ motif and the L1014F and M918T mutations associated with pyrethroid resistance (indicated by a green *) highlighted. (b) Sequence alignments of the S6 segments of aphids, fruitfly and human TTX-resistant (Na_v_1.5) and -sensitive (Na_v_1.4) channels. The inner selectivity filter sequences are highlighted (grey frames). Of particular note, in relation to TTX insensitivity in aphids, is the presence within DI of a non-aromatic asparagine rather than an aromatic (phenylalanine or tyrosine) residue (orange framed).

**Fig. 3:**
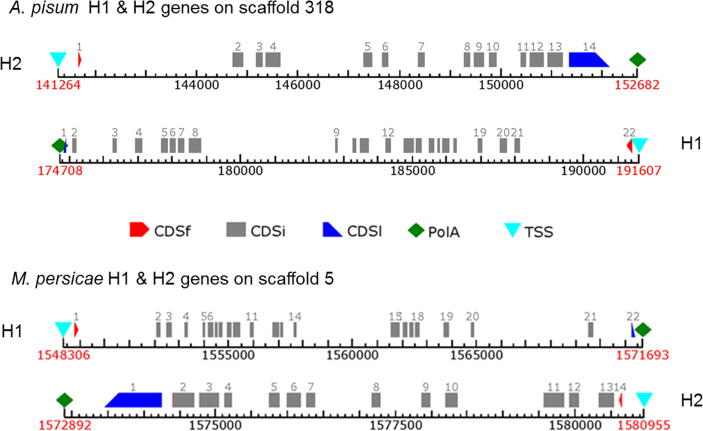
Genome organization. FGENESH (Softberry) gene predictions of the coding regions of the *A. pisum* (upper panel) and *M. persicae* (lower panel) channels on their genome scaffolds shows that, unlike other insects, aphid channels are encoded by two subunits. CDSf = first (starting with start codon) coding exon, CDSi = internal exon, CDSl = last coding segment, TSS = position of transcription start (TATA-box position), PolA = position of polyadenylation.

**Fig. 4:**
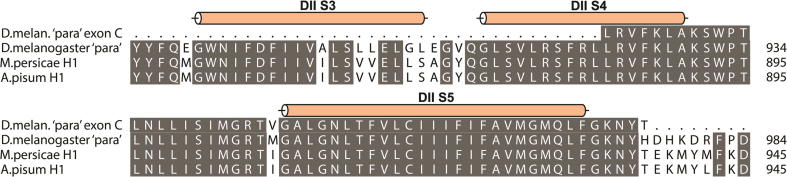
The mutually exclusive Drosophila ‘para’ c/d splice variants (DmNa_v_1 residues 923–976) are absent in aphid channels. Furthermore, within this highly conserved region of the channel, aphid channels have an isoleucine (an atc codon) at position 946 (numbering according to the DmNa_v_1 sequence), whereas in other insects (such as Drosophila) exon c has a valine (g/tn) and exon d a methionine (at/g), suggesting that the aphid channels may be derived from an ancestral Na_v_ lacking the exon duplication found in contemporary insect Na_v_1s.

The preliminary data described above suggested that the *A. pisum* Na_v_ channel is encoded by, and assembled from, two unique 2x6TM heteromers (Fig. 2a), here designated as H1 and H2. On closer analysis of the genomic data we established that the two putative genes are orientated in opposite directions on scaffold 318, separated by approximately 23 Kb of non-coding sequence (Fig. 3). H1 has two identifiable alternative exons corresponding to exons j and b in the *Drosophila melanogaster* DmNa_v_1 (para) gene [7], but unusually the mutually exclusive c/d splice variants (DmNa_v_1 residues 923–976 (Fig. 4)) are absent. Furthermore, within this highly conserved region of the channel, *A. pisum* has an isoleucine (an atc codon) at position 946 (numbering according to the DmNa_v_1 sequence), whereas in other insects exon c has a valine (g/tn) and exon d a methionine (at/g), suggesting that the *A. pisum* channel may be derived from an ancestral Na_v_ lacking the exon duplication found in contemporary insect Na_v_1s. Within H2, exons 6 and 7 correspond to the mutually-exclusive exons k/l in DIII of insect Na_v_1s (Supplementary Fig. 1).

**Supplementary Fig. 2:**
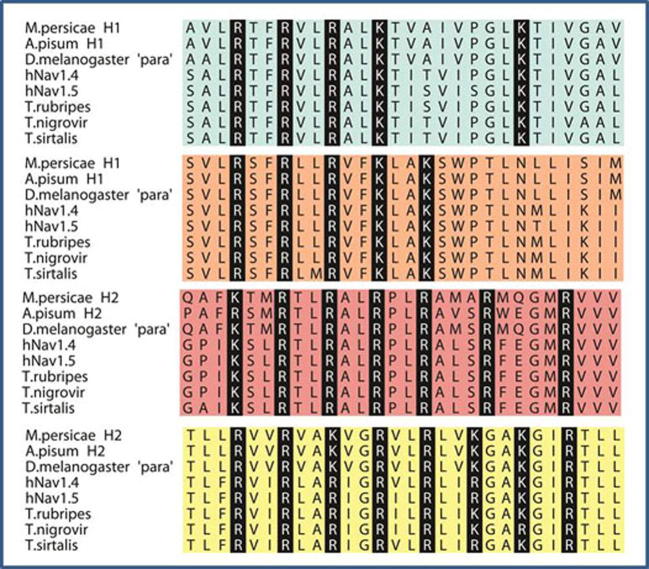
Alignment of S4 helices within Na_v_ channel voltage sensing domains. Conserved positively-charged residues [black highlights] in the S4 helices in the voltage sensing domains of the H1 (cyan and orange) and H2 (pink and yellow) aphid subunits and the DI (cyan), DII (orange), DIII (pink) and DIV (yellow) segments, respectively, of the non-aphid polypeptides.

**Supplementary Fig. 3:**
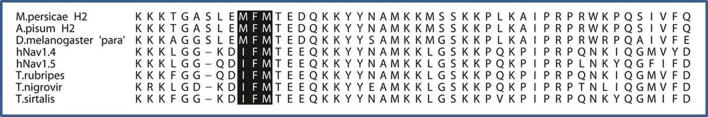
Alignment of the domain III–IV interconnecting loop region of Na_v_ channels. The inactivation particle (M/I) FM motifs in DI (H2 of aphids) and DIII (other species) are highlighted.

**Fig. 5:**
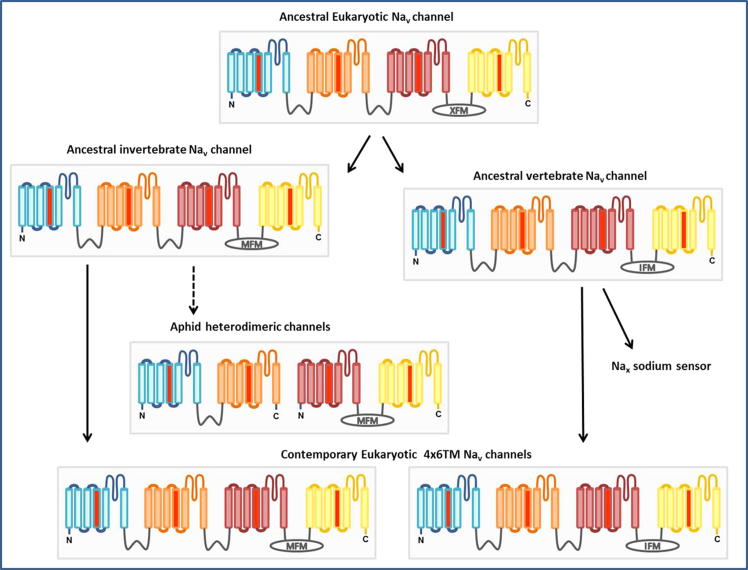
Scheme of the proposed evolutionary relationships between members of the eukaryotic Na_v_ channel family. The DIII-IV fast inactivation particle ‘MFM’ motif, unique to invertebrate Na_v_1s, is present in aphid H2 only, suggesting that H1 and H2 need to co-assemble to form a fully functional, multi-domain channel [12]. The presence of the conserved ‘MFM’ motif in aphid H2, along with the high degree of H1 and H2 sequence identity with contemporary insect 4x6TM Na_v_1 channels, suggests that the aphid heterodimeric assembly has arisen by functional modification of an ancestral 4x6TM invertebrate Na_v_ channel.

**Fig. 6:**
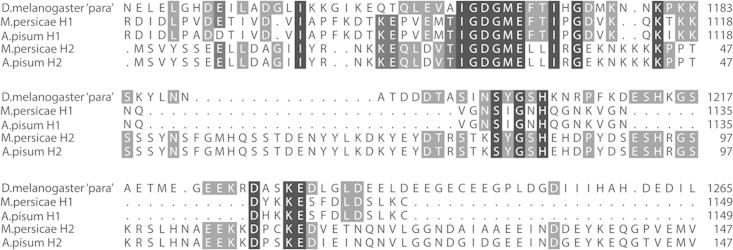
Proposed duplication of part of the domain II-III linker region of an ancestral Na_v_1, corresponding to exons 20 and 21 in H1, to give rise to exons 2 and 3 in H2, illustrated by the duplicated sequence motifs IGDGME and SXGXH(X)*_n_*D(X)_2_KE, highlighted in black.

To determine if the predicted heterodimeric organisation of the ApNa_v_1 is unique to *A. pisum*, we used degenerate PCR and RACE to amplify corresponding full-length Na_v_1 cDNAs from the closely related and agriculturally important pest aphid, *Myzus persicae* (Sulzer). The *M. persicae* channel was also found to be encoded by two genes (submitted NCBI Accessions FN601405 and FN601406), organized identically to those of the *A. pisum* gene prediction; the only exception being that the cDNA for H1 does not incorporate alternative exon j. The *M. persicae* and *A. pisum* H1 and H2 sequences are highly conserved at the amino acid level and have high (64% (DI–DII); 68% (DIII–DIV)) amino acid identity with equivalent domains in *D. melanogaster* DmNa_v_1 channels (Supplementary Fig. 1). For both aphids the heteromers encode the full set of positively charged residues in the S4 helices of the voltage-sensing domains required to sense changes in membrane potential and initiate channel activation (Supplementary Fig. 2). The conserved tripeptide ‘MFM’ motif, unique to invertebrate Na_v_1s, which forms the fast inactivation particle in the intracellular loop between DIII and DIV [10], is present in H2 only, as are the adjacent charged residues that modulate fast inactivation [11] (Supplementary Fig. 3), a strong indication that H1 and H2 need to co-assemble to form a fully functional, multi-domain channel [12]. The presence of a conserved ‘MFM’ motif in H2, along with the high degree of sequence identity of H1 and H2 to contemporary insect 4x6TM Na_v_1 channels, suggest that the aphid heterodimeric assembly has arisen by structural modification of an ancestral 4x6TM invertebrate Na_v_ channel (Fig. 5). We believe that this modification most probably occurred by gene fission [13]. Prior to the gene fission event it is likely that there took place a duplication of part of the domain II–III linker region in the ancestral gene, corresponding to exons 20 and 21 in H1 to give rise to exons 2 and 3 in H2, which may have triggered the gene fission. This can be seen in the duplication of the sequence motifs IGDGME and SXGXH(X)*_n_*D(X)_2_KE in this region (Fig. 6). Short novel bits of DNA (7 bp (exon 22 in H1) and 19 bp (exon 1 in H2) must have subsequently been acquired to provide start and stop codon sequences for H2 and H1, respectively. No comparable examples of 2-domain Na_v_s could be identified by BLAST searching of the available metazoan genomes in the NCBI database, suggesting that this is a rare evolutionary divergence which may be confined to aphids.

Many insect species, including important crop pests such as aphids, have evolved resistance to pyrethroid insecticides via specific amino acid substitutions within the Na_v_1 channel [14]. Further evidence supporting a heterodimeric Na_v_1 organization in aphids was obtained by amplification of full-length H1 cDNAs from pyrethroid-resistant *M. persicae* (Supplementary Table 2), using primers unique to the 5′ and 3′ termini of H1. This yielded H1 amplicons containing the classic DII L1014F and M918T amino acid substitutions associated with pyrethroid-resistance [8], demonstrating the amplification of a functional gene that has been subjected to recent evolutionary modification [15] rather than a pseudogene. Taken together all of this evidence strongly supports the view that aphids have a 4x6TM Na_v_ channel encoded by two genes.

The recent pre-release of the *M. persicae* genome (on AphidBase) further corroborates these findings, with both H1 and H2 sequences of *M. persicae* being identifiable on scaffold 5 of the version 2 assembly for clone G006. The two genes are again (as with the *A. pisum* channel) orientated in opposite directions on the genomic scaffold (Fig. 3), but this time in immediate (∼1 Kb) proximity to each other. Given this well-maintained gene organization in the aphid genomes, one could speculate that expression of these subunits may be controlled by regulatory elements such as enhancers which can work in a bidirectional manner [16], so the “back-to-back” orientation of the two genes may be highly instrumental in allowing the simultaneous co-expression of the two genes. Notably, the H1 prediction for clone G006 also contains both pyrethroid-resistance associated mutations [8].

For the aphid Na_v_1 channels the highly conserved P-loops that form the outer vestibule of the channel pore are identifiable. However, the sodium selectivity filter motif, formed by a single residue from each of the 4 domains, which is normally DEKA in Na_v_s, has an asparagine (N) in DIII and a serine (S) in DIV, producing a DENS selectivity filter (Fig. 2a and b). A precedent for a DENS-like selectivity filter motif in a Na_v_ is the DENA motif of the human Na*_x_* isotype, which putatively functions as a sodium sensor channel [17]. In most Na_v_s the positively charged DIII selectivity filter lysine is critical for channel function, stabilizing the pore via electrostatic interactions with the negatively charged DI aspartate, whereas its interaction with the negatively charged DII glutamate appears to provide a basis for the selective permeation of Na^+^ over K^+^ [18]. Electrostatic repulsion by the lysine side chain also promotes block of Ca^2+^ and other divalent cations [18,19]. Thus the DENS selectivity filter in the aphid channel may profoundly affect the ion selectivity of the channel due to the absence of the critical lysine residue (as described later). The DIII lysine in the Na_v_ selectivity filter is also integral to the binding of TTX [20]. Another principal TTX binding determinant is a DI aromatic phenylalanine or tyrosine residue, located adjacent to the aspartate of the selectivity filter [21], and substitution with a non-aromatic amino acid at this position accounts for TTX-insensitivity in the majority of tetrodotoxic animals [22–26]. Significantly, both aphid channel sequences have an asparagine at this position (Fig. 2b).

**Supplementary Fig. 4:**
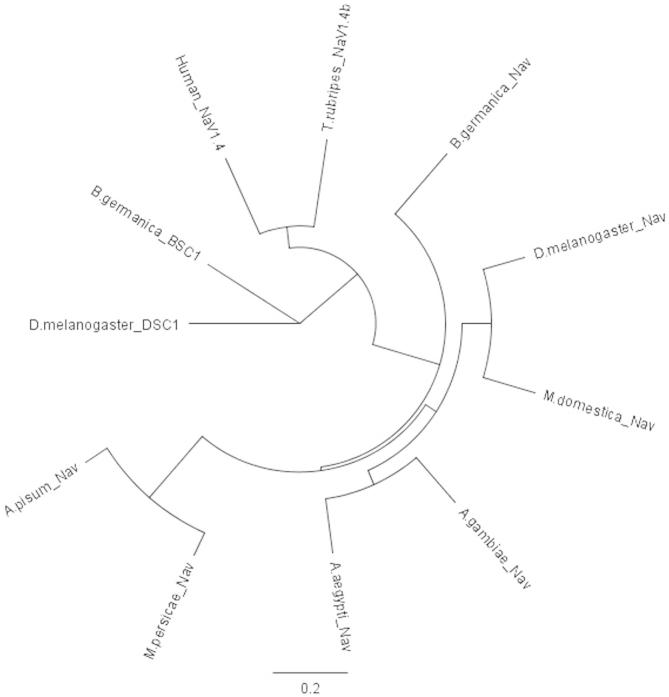
Na_v_1/Na_v_2 phylogenetic tree. DNA sequences were imported into BIOEDIT v 7.0.9.0 and converted to amino acid sequence for alignment with ProbCons v1.10. Pal2Nal was utilized to convert the amino acid sequence alignment and the corresponding DNA sequences back into a codon alignment. Codon alignments were subsequently imported into into Geneious (http://www.geneious.com/). A phylogenetic tree was constructed using Geneious Tree Builder with Tamura-Nei as the Genetic Distance Model and Neighbor Joining method, with the DSC1 sequence as outgroup, 1000 bootstrap replicates with random seed was employed to generate a Consensus tree with support threshold cut-off set at 80%.

**Fig. 7:**
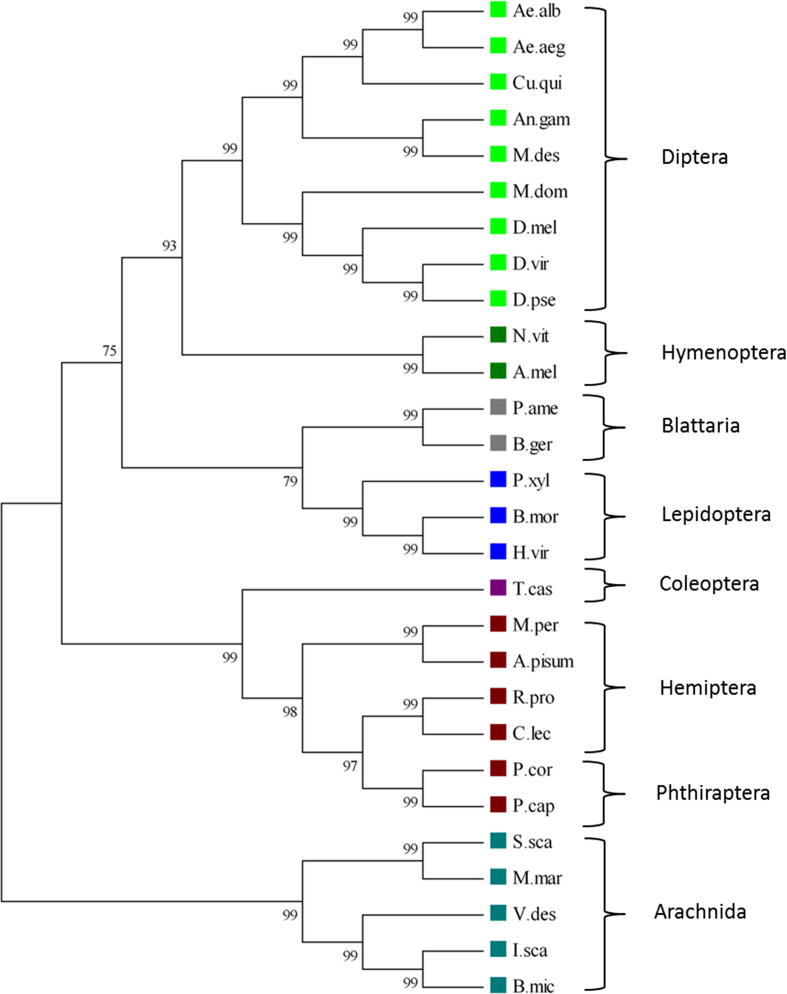
Maximum-likelihood tree of insect and arachnid Na_v_1 channel a-subunits. The best nucleotide substitution model (GTR) was selected by Topali v2 [41], based on codon alignment of Na_v_1 channels. PhyML 3.0 aLRT [42,43] gave estimated values for gamma shape parameters (0.421) and proportion of invariant sites (0.189). Na_v_1 channels from *Aedes albopictus*, *Aedes aegypti*, *Culex pipiens quinquefasciatus*, *Anopheles gambiae*, *Mayetiola destructor*, *Musca domestica*, *Drosophila melanogaster*, *Drosophila virilis*, *Drosophila pseudobscura*, *Nasonia vitripennis*, *Apis mellifera*, *Periplaneta americana*, *Blattella germanica*, *Plutella xylostella*, *Bombyx mori*, *Heliothis virescens*, *Tribolium castaneum*, *Myzus persicae*, *Acyrthosiphon pisum*, *Rhodnius prolixus*, *Cimex lectularius*, *Pediculus humanus corporis*, *Pediculus humanus capitis*, *Sarcoptes scabiei*, *Mesobuthus martensii*, *Varroa destructor*, *Ixodes scapularis*, *Boophilus (Rhipicephalus) microplus* are represented on the tree and are grouped phylogenetically. Branch confidence values above 60% are indicated. Aphids (Hemiptera) are estimated to have diverged from Phthiraptera and other insect classes around 172.6 MYA and 371.9 MYA respectively, and from the Arachnida approximately 581.8 MYA (www.timetree.org).

A phylogenetic analysis, to determine whether the aphid channels are more closely related to Na_v_1 (e.g. *D. melanogaster* ‘para’) or the sodium channel ancestor Na_v_2 (e.g. *D. melanogaster* DSC1 [27]), was crucial in order to understand the evolution of these intriguing aphid channels. Previous work has shown that because of their unique pore (DKEA or DEEA) Na_v_2 channels conduct calcium [4,28] and act to modulate the stability of neuronal circuits [29]. Our studies indicate that the channels identified in aphids typically cluster with Na_v_1 type channels (Fig. 7), rather than Na*_v_*2 channels (Supplementary Fig. 4), and that the aphid Na_v_1-like channels segregate with other hemipteran Na_v_1 channels on the tree. Interestingly, the DSC1 homologues identified in aphids are 4x6TM multi-domain channels [5], so the evolutionary modification described applies only to aphid Na_v_1 homologues.

**Fig. 8:**
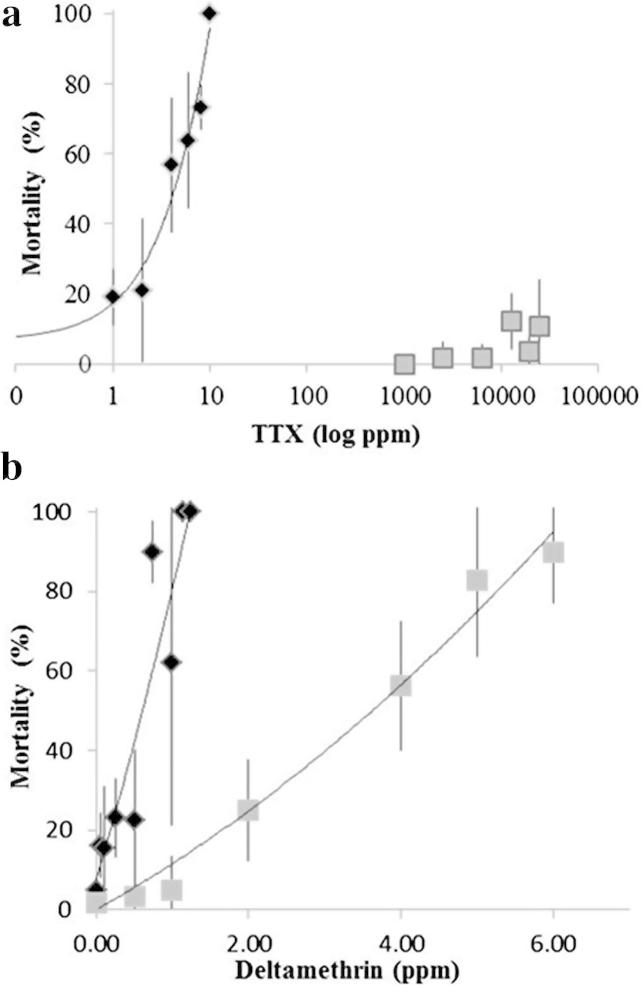
Bioassays with TTX and pyrethroid insecticides. (a) Immersion of aphids () in high concentrations (25 000 ppm (78 mM)) of TTX failed to cause significant mortality, whilst *D. melanogaster* (♦) treated with considerably lower doses were acutely affected (LD_50_ = 3.65 ppm (11.4 μM) TTX). Due to the total lack of toxic response, an LD_50_ for TTX on aphids could not be calculated. (b) Both species showed comparable toxic responses to a pyrethroid insecticide (deltamethrin): Drosophila LD_50_ = 0.63 ppm (0.01 μM) and aphid LD_50_ = 3.25 ppm (0.07 μM). Data points represent the average of three replicate tests. Error bars represent the standard deviation (S.D.) for each test point.

**Fig. 9:**
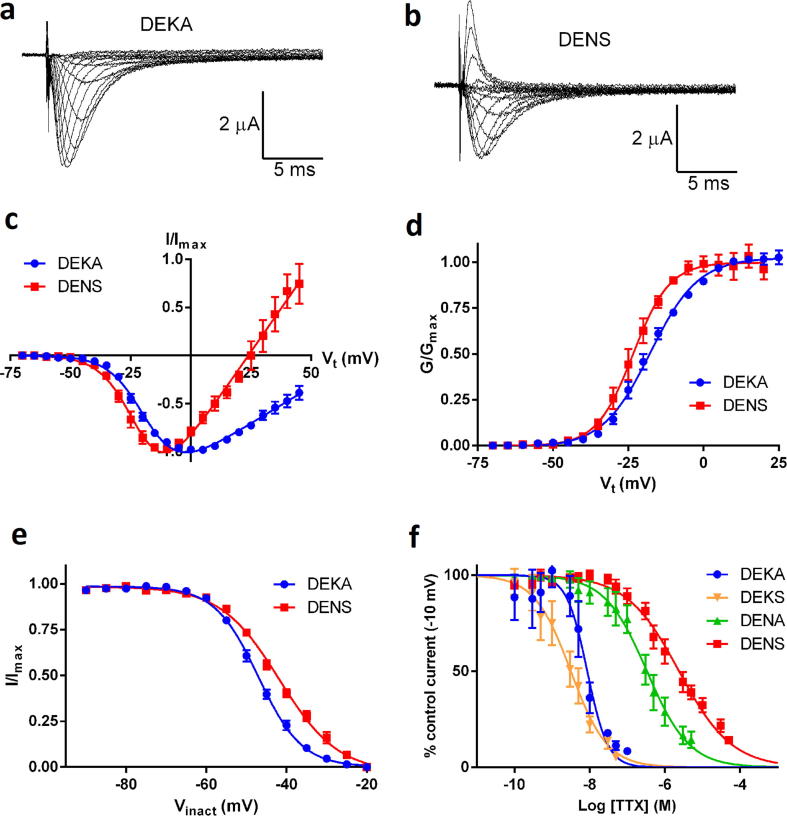
Electrophysiological characterization of WT DmNa_v_1 and DEKA mutant Na_v_1 channels expressed in Xenopus oocytes. (a) and (b) Ionic currents evoked by stepping the membrane potential from −70 mV to values in the range −70 mV to +45 mV for DEKA (a) and DENS (b) channels. Shown are traces for −50 mV to −15 mV in 5 mV increments and −15 mV to +45 mV in 10 mV increments respectively. (c) Current–voltage relationships for the DEKA and DENS channel. Currents were activated by holding the oocytes at −70 mV and stepping the voltage to values in the range −70 to + 45 mV in 5 mV increments. Data points are mean normalized current (*I*/*I*_max_) ± S.E.M. and have been fitted by a Boltzmann-IV equation. The reversal potentials were 71.4 ± 2.7 mV (*n* = 16) for the DEKA channel and 25.1 ± 0.7 mV (*n* = 8) for DENS. (d) Conductance–voltage relationships for the DEKA and DENS channels. Data points are mean normalized conductance (*G*/*G*_max_) ± S.E.M. plotted against test potential and have been fitted by a Boltzmann equation. The half maximal activation potentials were −17.6 ± 0.5 mV for DEKA and −23.4 ± 0.7 mV for DENS. (e) Voltage dependence of steady-state inactivation of DEKA and DENS channels. Channels were exposed to inactivating pre-potentials in the range −90 mV to −20 mV in 5 mV increments followed by a test potential of −10 mV. Data points are mean normalized current (*I*/*I*_max_) ± S.E.M. in response to the -10 mV test potential plotted against the inactivating pre-potential. The curve-fit is to a Boltzmann equation giving half maximal inactivation potentials of −47.0 ± 0.3 mV (*n* = 11) for the DEKA channel and −41.8 ± 0.5 mV (*n* = 13) for the DENS channel. (f) Concentration-inhibition curves for TTX block of DEKA mutant channels. Channel current was evoked by holding the oocytes at −70 mV and stepping the voltage to −10 mV for 35 ms. Data points are mean% of control current (no TTX) ± S.E.M. and have been fitted by a four parameter logistic equation. IC_50_s were: DEKA, 8.67 ± 1.23 nM (*n* = 7); DEKS, 3.17 ± 0.65 nM (*n* = 10); DENA, 335 ± 47 nM (*n* = 4); DENS, 2393 ± 262 nM (*n* = 11).

**Fig. 10:**
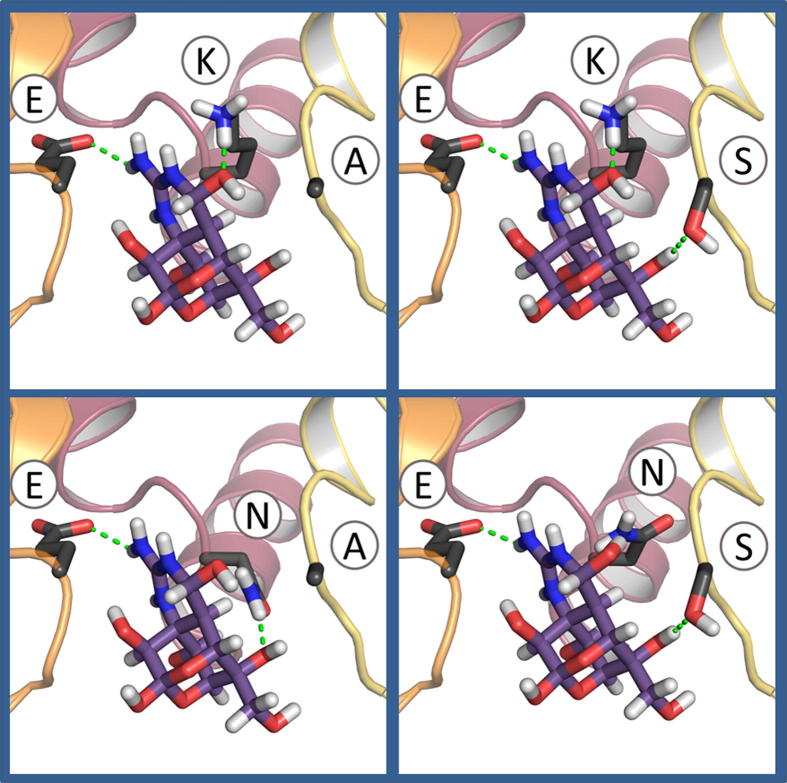
Models of the selectivity filter regions of unmodified and modified *D. melanogaster* DmNa_v_1 channels shown in ribbon representation with docked TTX and selectivity filter side chains in stick representation. Green dashes depict hydrogen bonds and the salt bridge between the TTX guanidinium moiety and DII glutamate (E) side chain. The wild-type (DEKA) motif and modified (DEKS, DENA, and DENS) motifs are shown. Different interactions with TTX are present in each model, with the seemingly important K-TTX interaction missing in DENA and DENS mutants which lack TTX sensitivity (Table 1, Supplementary movies 1–5). Domain I is not depicted for clarity.

Further evidence that no conventional TTX-sensitive Na_v_1 channel is present in aphids was obtained experimentally by determining the sensitivity of aphids to TTX. Bioassays of *M. persicae* with high concentrations of TTX (25 000 ppm, 78 mM) resulted in little mortality with the aphids being >2500-fold less susceptible to the toxin than were *D. melanogaster* (Fig. 8). Thus the Na_v_1 homologue in aphids must be highly insensitive to TTX, ruling out the presence of a conventional TTX-sensitive Na_v_1. The contributions of the aphid DIII (K → N) and DIV (A → S) selectivity filter substitutions to TTX insensitivity were also tested directly using heterologous expression and two-electrode voltage clamp (Table 1, Fig. 9f). Because the aphid channels did not express in oocytes, we used *D. melanogaster* DmNa_v_1 as a platform for analysis of the aphid channel’s selectivity filter. Three permutations of the selectivity filter, DEKS, DENA and DENS were introduced into the TTX-sensitive DmNa_v_1. The wild-type DEKA channels were highly susceptible to block by TTX (IC_50_ = 8.7 nM), the DEKS channels were marginally more sensitive, the DENA filter reduced the sensitivity by 38-fold (consistent with the findings of Penzotti et al. [20]), whereas the aphid sequence DENS was 275-fold less sensitive. We have further modelled the interaction of the aphid Na_v_1 P-loops with TTX *in silico* (Fig. 10). Our pore model, based on the P-loop region of the bacterial Na^+^ channel NavAb [30], differed somewhat from a previous TTX binding model of Fozzard & Lipkind [31] that was developed from homology with potassium channels prior to the availability of any sodium channel crystal structures (for a detailed comparison see Supplementary data 1). In our model, TTX docked with the unmodified DEKA channel shows classic interactions with known TTX-binding determinants, including D377 [20] and F378 [21] (housefly numbering) in DI, E985 (DII) [20] and K1497 (DIII). These observations agree with those of Penzotti et al. [20], who suggested that the K1497 extended side-chain underpins and stabilizes the TTX-bound state. A predicted sensitivity to TTX in the order: DEKS (GoldScore 67.9) > DEKA (59.4) > DENS (51.5) > DENA (44.3) was calculated from docking simulations, in good agreement with the IC_50_ values obtained in the electrophysiology experiments (Table 1). The presence of an additional hydrogen bond between the DIV serine and TTX in the DEKS model (Fig. 10) may provide a molecular basis for the increased TTX sensitivity of the DEKS channel over the DEKA channel. We conclude that the high level of insensitivity to TTX in aphids is principally due to the absence of an aromatic residue adjacent to the selectivity filter residue in DI, with the effect enhanced by the DIII lysine-to-asparagine substitution within the channel filter. We were, however, unable to detect any fingerprints of positive selection for TTX-insensitivity in the aphid channels using comparative analysis of multiple Na_v_1 sequence alignments and several codon-based maximum likelihood methods to estimate the rates of non-synonymous vs synonymous (dNdS) substitution (also known as Ka/Ks) ratios at each codon.

Amino acid substitutions within the P-loops of Na_v_s that decrease TTX-binding also invariably reduce Na^+^ permeability or Na^+^ selectivity [23]. Our electrophysiological experiments that introduced the DENA and DENS selectivity filters into DmNa_v_1 revealed large shifts in reversal potential (*V*_rev_) compared to DEKA (Table 1, Fig. 9c) as well as the appearance of tail currents. This indicates a compromised selectivity for Na^+^ and (consistent with observations of Favre et al. [18]) an increase in Ca^2+^ permeability possibly causing the activation of Ca^2+^-activated Cl^-^ currents. However, it should be noted that these results were obtained using a limited number of modifications on a DmNa_v_1 template, and other critical (as yet unidentified) differences in residues within the pore region of the aphid Na_v_1 channels (Fig. 2b) may play an important role in determining the channels actual ion selectivity and conductance properties.

## 3 Discussion

We have identified a unique heterodimeric voltage-gated Na_v_1-type channel in aphids which may have arisen through evolutionary adaptation (fission) of a monomeric 4x6TM invertebrate Na_v_ channel ancestor. The alternative scenario of a gene duplication followed by two alternative domains being lost individually in two duplicated genes which then reconstitute a functional channel is not at present well supported. Evidence to substantiate the feasibility of a two subunit Na_v_1 channel has previously been demonstrated by the ability of two split poly-peptides to reconstitute sodium channel function [12]. The modified selectivity filter of the aphid channels also raises questions about how aphids propagate and maintain action potentials along their neurons.

The possibility that aphids have an evolutionary adaptation whereby propagation of action potentials is no longer exclusively due to sodium is intriguing. It has been shown previously that, compared with other insects, aphids maintain a uniquely low concentration of sodium (0.2–2 mM) and calcium (2 mM) in their haemolymph [32,33]_,_ which may be related to their phloem feeding. The levels of Na^+^ (and Ca^2+^) concentrations recorded are unlikely to support conventional action potentials unless highly effective ion-exchange barriers are in place to concentrate these ions in the neuronal micro-environment; there is however no definitive anatomical evidence to support this.

It is notable that other closely related phloem-feeding hemiptera such as white fly (e.g. *Trialeurodes vaporariorum*) have fully TTX-sensitive Na_v_s [34]. This suggests that the evolutionary driver for Na_v_1 channel adaptation in aphids was not the low sodium concentrations encountered in the phloem sap, but possibly the necessity for acquiring insensitivity to a TTX-like molecule. In most cases where TTX is found in vertebrates, it is produced by symbiotic bacteria, or taken up from the food chain by consuming TTX-accumulating organisms [22]. Significantly, many aphids have bacterial symbionts that may have a facultative role in protecting them from environmental stresses and predators [35]. One such symbiont, *Serratia symbiotica*, is closely related to free-living members of the same genus, including *Serratia marascens* which is capable of producing TTX. However, despite the possibility of previous exposure to TTX (or a TTX-like molecule) being a credible hypothesis for the evolution of a TTX-insensitive channel in aphids, we were unable to detect any fingerprints of positive selection for TTX-insensitivity in the aphid channels on comparative analysis of multiple Na_v_1 sequence alignments using several codon-based maximum likelihood methods [36]. So the likelihood is that the TTX-insensitivity in aphid Na_v_1 channels is purely due to neutral evolution or the result of genetic drift. Clearly there is still much work to be done to understand the basis of the adaptations described and the true nature of neuronal signalling in aphids.

## 4 Methods

*Insect strains: M. persicae* clone 4106A is a laboratory-reared clone fully susceptible to insecticides and originating from potatoes in Scotland in 2000. 2169G is a clone initiated from a field collection from Brussels sprouts in Lincolnshire, England in 1997, highly resistant to pyrethroid insecticides. Post-collection, both aphid clones have been maintained on Chinese cabbage leaves in the insectary at Rothamsted. Clone G006 used for genome sequencing was collected from pepper plants in Geneva, New York in 2003.

*Molecular biology:* DNA sequence data was downloaded from AphidBase, http://www.aphidbase.com/aphidbase/. Aphid genomic scaffolds harbouring Na_v_-like sequences were identified using TBLASTN homology searching with the *D. melanogaster* DmNa_v_1 sequence. Gene predictions were facilitated using FGENESH software (www.softberry.com).

Total RNA was extracted from aphids (*M. persicae*) snap frozen with liquid N_2_ using Trizol reagent (Invitrogen). PCR was used to amplify and clone two cDNA fragments corresponding to ∼90% of DI–DII and DIII–DIV of the *M. persicae* channel. cDNA synthesis used 5 μg of freshly isolated total RNA and enhanced Avian reverse transcriptase (Sigma) at 50 °C with an oligo-δT_20_V primer. PCR was performed initially with degenerate primers (Supplementary Table 3) complementary to the predicted channel sequence of *A. pisum* and subsequently with specific primers (Supplementary Table 3) and PFU enzyme (Promega). Rapid amplification of cDNA ends (RACE) was used to obtain the sequences of the termini of both subunits using a RLM-RACE kit (Ambion); this enabled the unambiguous identification of the start and stop sequences for H1 and H2 and the first exon of H2. Thermo-cycling conditions were: 95 °C × 2 min, then 35 cycles of: 95 °C × 20 s, 50 °C × 20 s, 72 °C × 6 min, with a final elongation of 92 °C × 12 min. PCR products were sequenced either directly or once cloned using BigDye v1.1 (ABI). Full-length cDNAs were PCR amplified using specific primers and a proof-reading polymerase.

The *D. melanogaster* DmNa_v_1 cDNA inserted in a pGH *Xenopus* expression vector [37] was used as the template for the creation of selectivity filter mutants. Site-directed mutagenesis used the QuikChange SDM kit (Stratagene) to generate K1497N and A1790S single mutations and a K1497N plus A1790S double mutant (housefly numbering; Supplementary Table 1). All constructs were verified by DNA sequencing. Bacterial cultures of channel-containing plasmids were maintained at 30 °C to minimise plasmid rearrangements. The mMESSAGE mMACHINE kit with T7 promoter (Ambion) was used for cRNA synthesis from plasmid linearized with *Not*I.

*Phylogenetic tree construction:* DNA sequences were imported into BIOEDIT v 7.0.9.0 [38] and converted to amino acid sequences for alignment with ProbCons v1.10 [39]. PAL2NAL [40] was used to convert the amino acid sequence alignment and the corresponding DNA sequences back into a codon alignment. Codon alignments were subsequently imported into BIOEDIT and manually edited to remove gaps and regions of poor homology. TOPALi v2 [41] was used for phylogenetic model selection, and PhyML 3.0 aLRT [42,43] for Maximum-likelihood and Shimodaira-Hasegawa-like Approximate-Likelihood ratio based tree construction, with GTR nucleotide substitution model, 6 substitution rate categories and estimated values for transition/transversion ratio, gamma shape parameters and proportion of invariant sites. Trees were saved in .nwk format and visualized and edited in BIOEDIT.

*Bioassays:* Ten 3rd or 4th instar apterous aphids (*M. persicae*, susceptible clone 4106A) were transferred onto leaf disks excised from Chinese cabbage (*Brassica rapa* var chinensis) and kept hydrated on 15 ml agar plugs in individual plastic pots with vented lids. 24 h later a dilution series was made of technical grade deltamethrin (Rothamsted Research) or TTX (Sigma Life Sciences) in wetting agent (0.01% v/v Agral) and the aphids were immersed in the solution for 4 s, removed and blotted dry on tissue paper before being returned to their leaf disk. Drosophila treatment was similar except that they were immobilized with CO_2_ before immersion and were kept post treatment in vented glass vials with 300 μl semi-defined Drosophila media set on the vertical wall of the vial. Insects were checked at 2, 18, 24, 38, 48, 72 and 96 h post treatment and scored as healthy, affected or dead. Those scored as “affected” at 48 h died by 72 h whereas those scored as alive survived the full time course. Analyses were performed by grouping the affected and dead insects at 48 h. There were 3 replicates for each concentration of ligand and each bioassay was repeated 3 times. LD_50_ values were calculated following Probit analysis [44].

*Electrophysiology:* Preparation, injection and culture of oocytes as well as the voltage clamp procedures, protocols and analyses are detailed in Burton et al. [45]. The composition of the external recording solution was: 95 mM NaCl, 2 mM KCl, 2 mM CaCl_2_, 1 mM MgCl_2_, 5 mM HEPES, pH 7.5. Some experiments were carried out in physiological media with a range of different external ion concentrations, including a low sodium/high potassium medium reminiscent of aphid saline, showing that robust inward currents were produced (data not shown). For TTX block experiments, untreated injected oocyte recordings were obtained in order to ascertain the presence of cation channels in the oocyte membrane then oocytes were equilibrated in the presence of TTX for 1 min before channel recordings were made. Incremental concentrations of TTX were added to the same oocyte until complete channel block was observed or until loss of oocyte viability. IC_50_ values for TTX inhibition were estimated by fitting concentration-inhibition data by a four parameter logistic equation using Graphpad Prism 6.0.

*Homology modelling:* A homology model of the *D. melanogaster* DmNa_v_1 selectivity filter was generated using the crystal structure of the bacterial sodium channel NavAb as template (PDB code 3RVY) [30] in MODELLER9v10 [46]. DeepView software [47] was employed to modify residues to produce the DEKS, DENA or DENS variants of the *D. melanogaster* selectivity filter. Automated docking predictions of TTX [48] (Cambridge Structural Database reference TETXHB) in a 12 Å radius site centrally positioned in the selectivity filter of the DmNa_v_1 models were generated using GOLD Version 5.1 [CCDC, Cambridge, UK]. For a more detailed description of the modelling methodology see Supplementary Materials and Methods.

*dNdS (Ka/Ks ratio) analyses:* Comparative analysis of sequence alignments using state-of-the-art statistical models (http://www.datamonkey.org/) was employed to analyse the Na_v_1 sequences for signatures of positive selection. Four different codon-based maximum likelihood methods, SLAC, FEL, REL [49], and FUBAR [50], were used estimate the *dN*/*dS* (also known as Ka/Ks or *ω*) ratio at every codon in the alignment. Each programme was run with optimised substitution model selection.

## Author information

The authors declare no competing financial interests. Correspondence should be addressed to T.G.E.D. (emyr.davies@rothamsted.ac.uk).

## Figures and Tables

**Table 1:** Effects of aphid selectivity filter residue changes to *D. melanogaster* DmNa_v_1 channels expressed in *Xenopus* oocytes: DENA and DENS mutants impart TTX-insensitivity and alter channel activity.

	Selectivity filter Motifs			
	DEKA	DEKS	DENA	DENS
*V*_rev_ (mV)	71.4 ± 2.7	83.9 ± 4.2^∗∗^	18.6 ± 0.6^∗∗∗^	23.9 ± 0.7^∗∗∗^
*V*_50,act_ (mV)	−17.6 ± 0.5 (16)	−14.3 ± 0.5 (12)	−12.6 ± 3.0 (8)^∗^	−23.4 ± 0.7 (8)
*k*_act_ (mV)	8.0 ± 0.5	9.6 ± 0.5	12.8 ± 2.2	6.1 ± 0.6
*V*_50,inact_ (mV)	−47.0 ± 0.3 (16)	−47.1 ± 0.6 (12)	−41.1 ± 1.5 (8)^∗∗∗^	−41.8 ± 0.5 (8)^∗∗∗^
*k*_inact_ (mV)	5.4 ± 0.3	5.2 ± 0.5	9.4 ± 1.2	7.4 ± 0.5
*τ*_decay_ – 10 mV (ms)	1.3 ± 0.03 (16)	1.4 ± 0.02 (12)^∗∗^	1.9 ± 0.1 (8)^∗∗∗^	1.8 ± 0.05 (8)^∗∗∗^
TTX IC_50_	8.7 ± 1.2 nM (7)	3.2 ± 0.6 nM (10)^∗∗∗^	335 ± 47 nM (4)^∗∗∗^	2393 ± 262 nM (11)^∗∗∗^
Size of current (at −10 mV)	>1 μA	>1 μA	>1 μA	>1 μA
Tail currents present	No	No	Yes	Yes

Statistical comparisons were made between the DEKA channel and the three pore variants using one-way ANOVA with Dunnett’s post-test. IC_50_ values were compared using an Extra Sum of Squares *F* test. Probability (*P*) values are ^∗^*P* *<* 0.05; ^∗∗^*P* < 0.01; ^∗∗∗^*P* < 0.001. Numbers in parentheses indicate the number of oocytes tested.

## Appendix A Supplementary data

Supplementary video 1 Aphid_TTX.

Supplementary video 2 Drosophila_TTX.

Supplementary video 3 Drosophila_DEKS_TTX.

Supplementary video 4 Drosophila_DENA_TTX.

Supplementary video 5 Drosophila_DENS_TTX.

Supplementary Table S1 Cross-species residue numbering.

Supplementary Table S2 Pyrethroid resistant *M. persicae* strains

Supplementary Table S3 Nucleotide sequence of primers used to amplify the entire coding sequences of HI and HII from *M. persicae*.
