# Supplementary figures and images for: An evolutionarily-unique heterodimeric voltage-gated cation channel found in aphids
Source: FEBS Lett. 2015 Feb 27;589(5):598–607. doi: 10.1016/j.febslet.2015.01.020 (PMC4332693; doi:10.1016/j.febslet.2015.01.020)

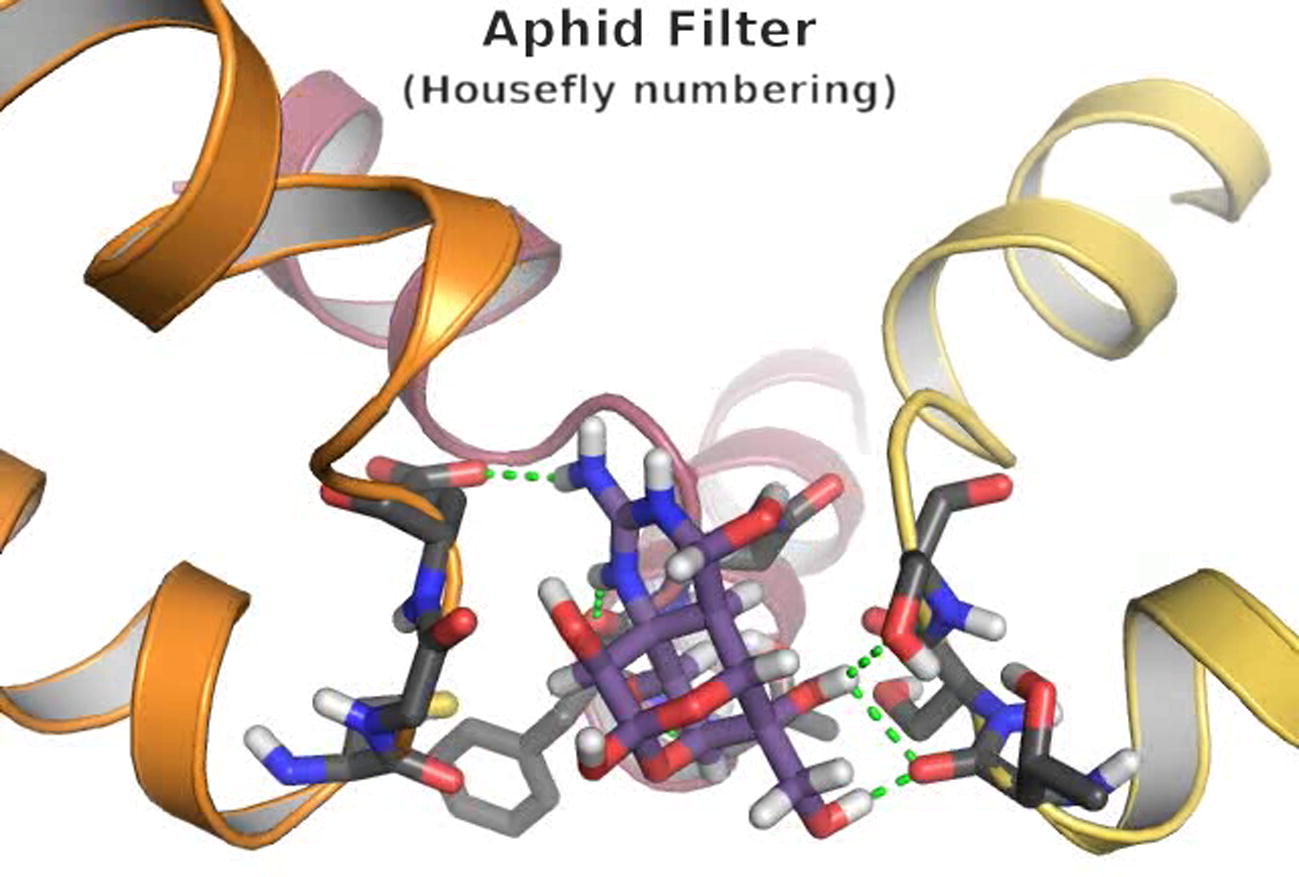

Supplement: Supplementary video 1 — Aphid_TTX. [file mmc1.jpg]

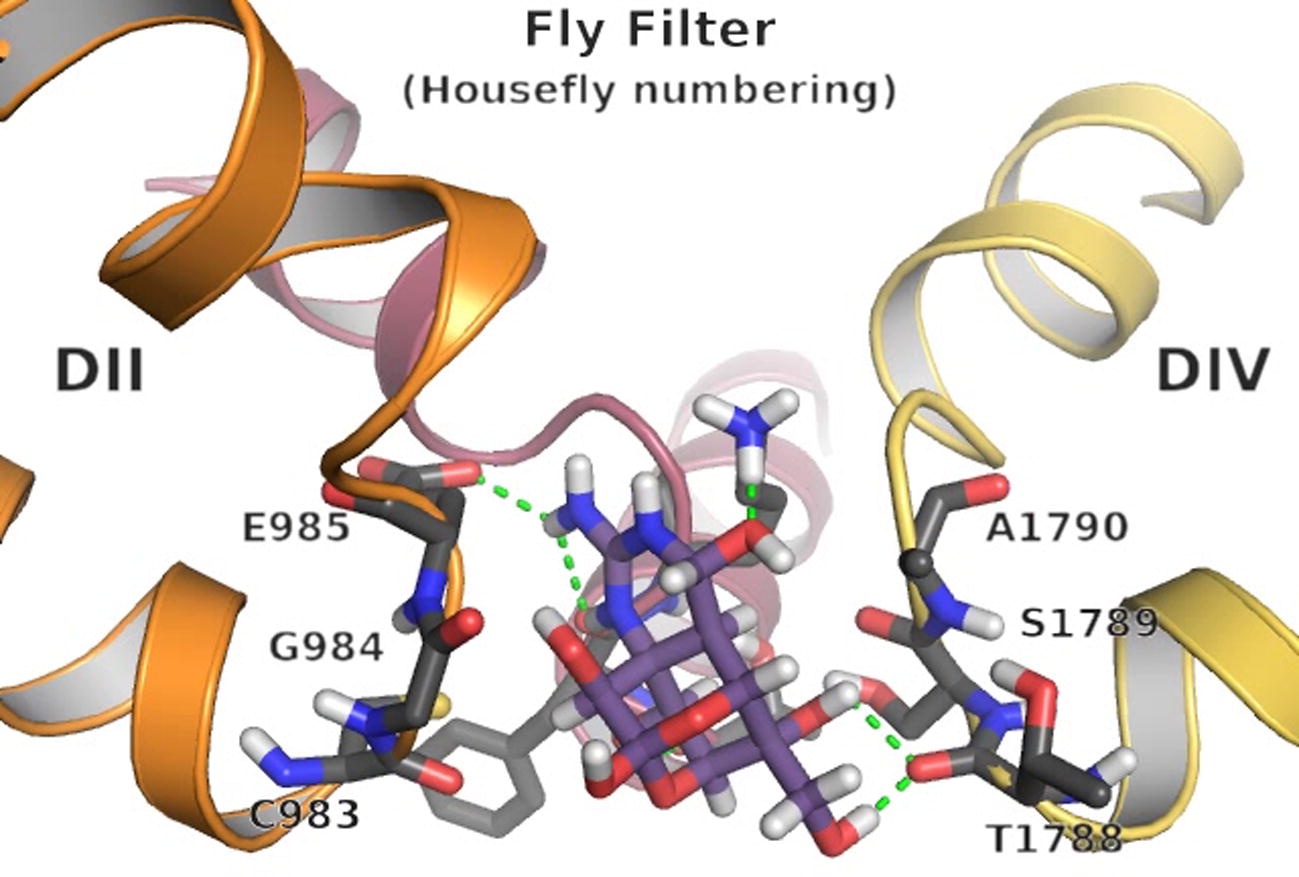

Supplement: Supplementary video 2 — Drosophila_TTX. [file mmc2.jpg]

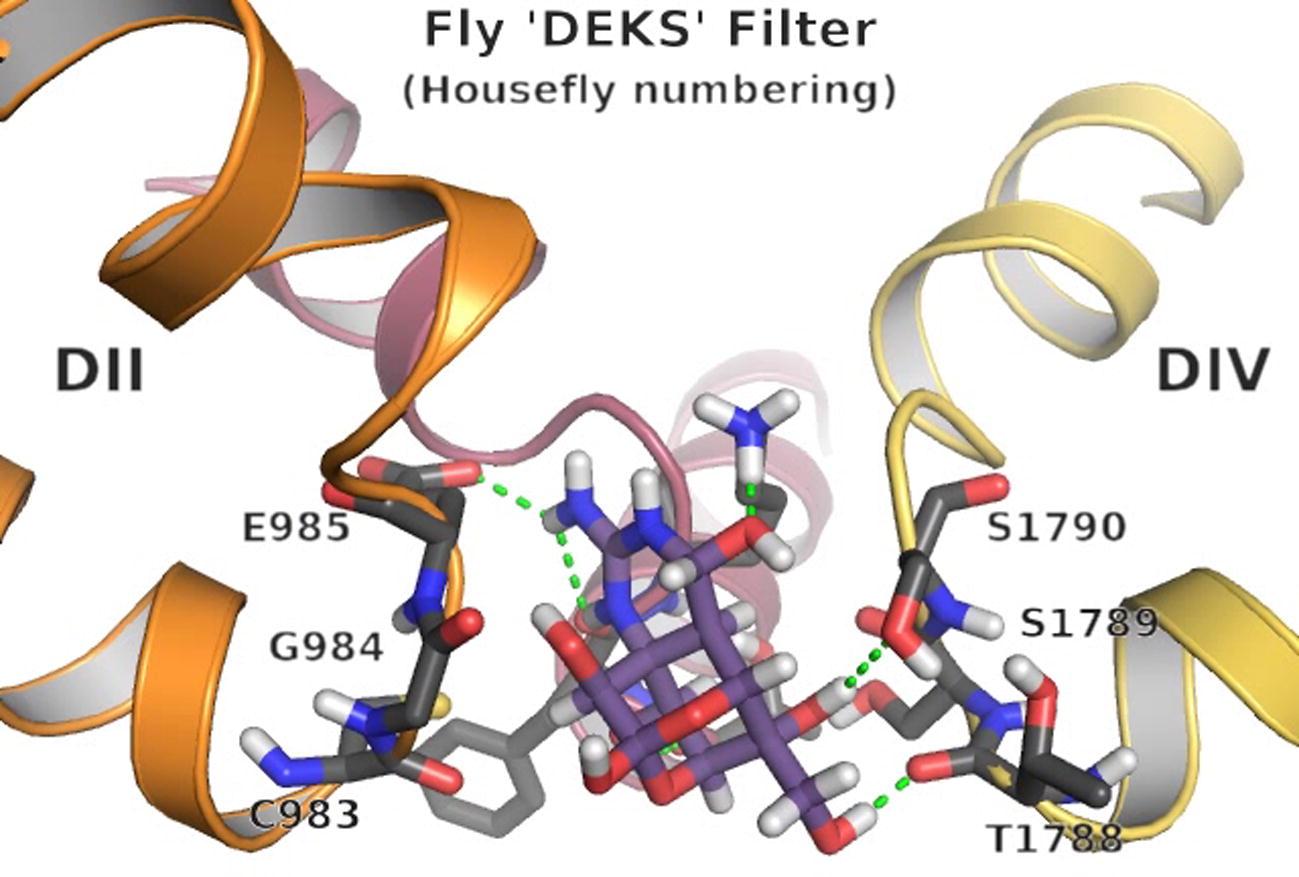

Supplement: Supplementary video 3 — Drosophila_DEKS_TTX. [file mmc3.jpg]

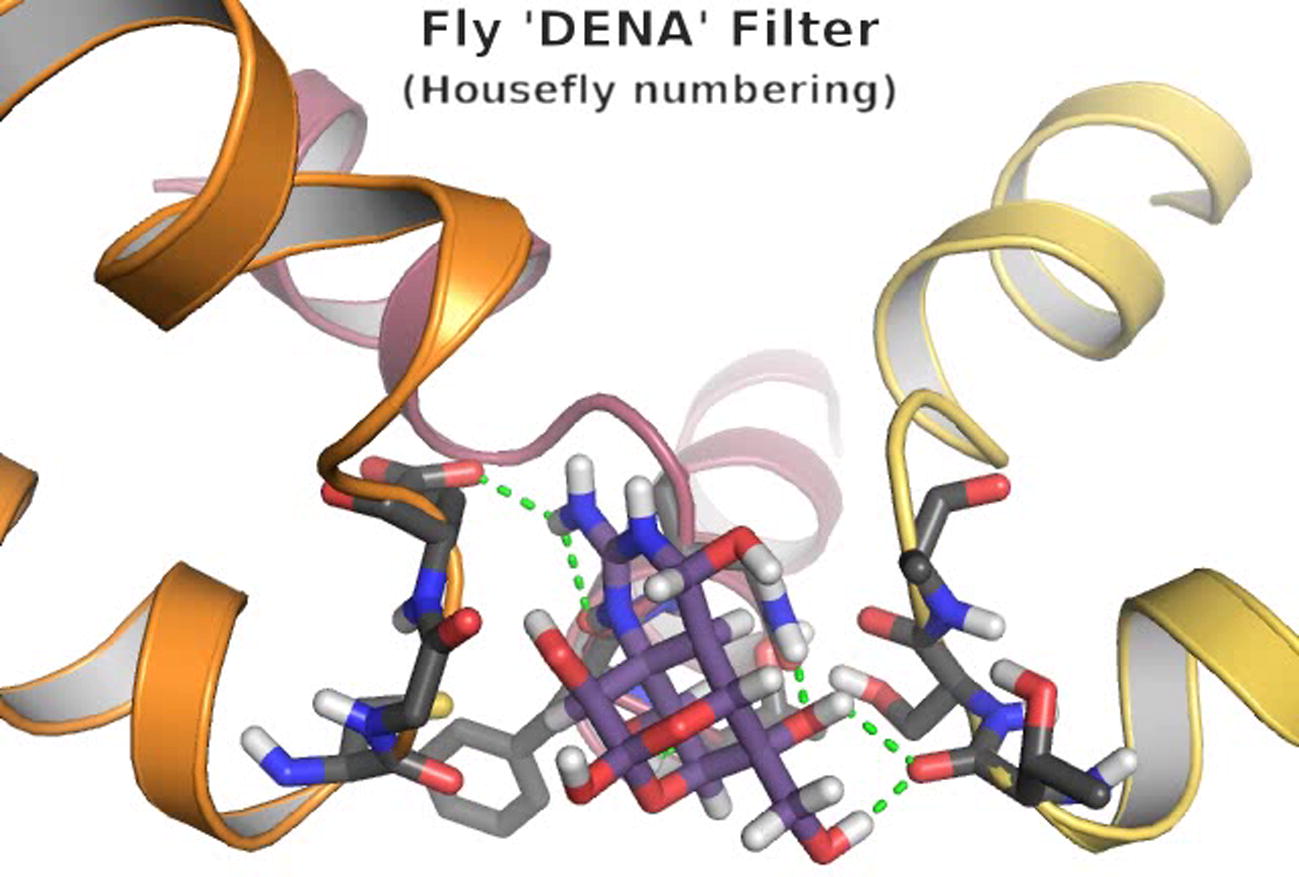

Supplement: Supplementary video 4 — Drosophila_DENA_TTX. [file mmc4.jpg]

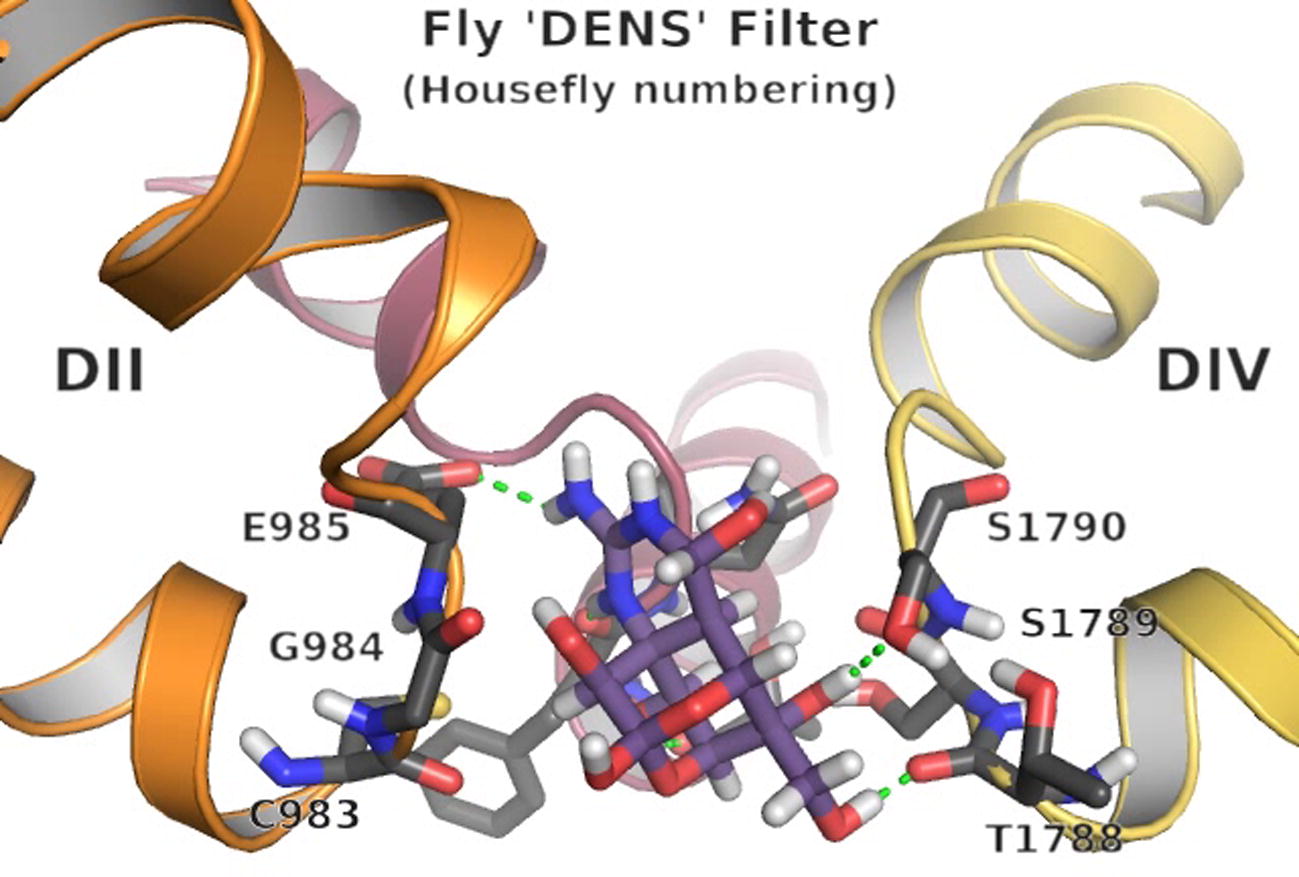

Supplement: Supplementary video 5 — Drosophila_DENS_TTX. [file mmc5.jpg]
