# Supplementary material for: An evolutionarily-unique heterodimeric voltage-gated cation channel found in aphids
Source: FEBS Lett. 2015 Feb 27;589(5):598–607. doi: 10.1016/j.febslet.2015.01.020 (PMC4332693; doi:10.1016/j.febslet.2015.01.020)
Supplement: Supplementary Table S1 — Cross-species residue numbering. [file mmc6.doc]

**Supplementary Table 3**

Nucleotide sequence of primers used to amplify the entire coding sequences of H1 and H2 from *Myzus persicae*.

| **Primer use** | **Forward primer** | **Reverse primer** |
| --- | --- | --- |
| Subunit H1 amplification | ATGTCCATTGCTGACACCGATTCTTC | CTAGCATTTTAAACTATCCAGGTCGAAACTT |
| Subunit H2 amplification | ATGAGTGTGTACAGTAGTGAGGAACTC | GACGTCTGCGAGTCTTGAGCT |
| Subunit H1 internal primers | ACCATGTCHATWGCTGACACCGATTC | TCCTTGATGATTTCCGATCGAA |
| Subunit H2 internal primers | CAGTAGTGAGGAACTCCTGGACG | AGCGGCCGCTTTAGACGTCGGCAAGTCTTGAAC |
| H1 5’ RACE primer |  | AGG GTT GAA GGG GTC GAG TGC  Nested with  CGT CGG TGG CGC TGA ACC T |
| H1 3’RACE primer | GCGCGAAGTTTATTGCGCAA |  |
| H2 5’RACE primer |  | CGG TCG GTG CTG GAG GTG TAC  Nested with  GCC AGT GCC AGA CTG CTA AGT A |
| H2 3’RACE primer | CGG ACG ACG ATT ACG ACA TGT ACT  Nested with  CTTCGACCCCAACGGCACCA |  |
