# Supplementary material for: An evolutionarily-unique heterodimeric voltage-gated cation channel found in aphids
Source: FEBS Lett. 2015 Feb 27;589(5):598–607. doi: 10.1016/j.febslet.2015.01.020 (PMC4332693; doi:10.1016/j.febslet.2015.01.020)
Supplement: Supplementary Table S2 — Pyrethroid resistant M. persicae strains [file mmc7.doc]

**SupplementaryTable 1 Cross-species residue numbering.**

|  | **Housefly reference sequence** | **Drosophila *para*** | ***M.persicae* H1** | ***M.persicae* H2** | **Human Na_v_1.4** | **Human Na_v_1.4** |
| --- | --- | --- | --- | --- | --- | --- |
| **Sequence IDs** | **(UniProt Q94615)** | **(UniProt P35500)** | **(NCBI FN601405)** | **(NCBI FN601406)** | **(TTX literature)** | **(UniProt** **P35499)** |
| Filter residue | D377 | D388 | D379 |  | D400 | D406 |
| Filter residue | F378 | F389 | N380 |  | Y401 | Y407 |
| Pyrethroid determinant | M918 | M933 | M903 |  |  | I693 |
| Filter residue | E985 | E1000 | E970 |  | E755 | E761 |
| Pyrethroid determinant | L1014 | L1029 | L999 |  |  | L791 |
| Filter residue | K1497 | K1509 |  | N406 | K1237 | K1244 |
| Filter residue | A1790 | A1802 |  | S700 | A1529 | A1536 |
