# Supplementary material for: An evolutionarily-unique heterodimeric voltage-gated cation channel found in aphids
Source: FEBS Lett. 2015 Feb 27;589(5):598–607. doi: 10.1016/j.febslet.2015.01.020 (PMC4332693; doi:10.1016/j.febslet.2015.01.020)
Supplement: Supplementary Table S3 — Nucleotide sequence of primers used to amplify the entire coding sequences of HI and HII from M. persicae. [file mmc8.docx]

**Supplementary Table 2** Pyrethroid resistant *M. persicae* strains

| **Strain** | **Pyrethroid Resistance status** | **Kdr** | **skdr** |
| --- | --- | --- | --- |
| 4106A | Susceptible | no | no |
| 794JZ | resistant | L1014F | no |
| 4824J | highly resistant (double homozygote) | L1014F | M918T |
| 2169G | highly resistant (double heterozygote) | L1014F | M918T |
