# Supplementary material for: An evolutionarily-unique heterodimeric voltage-gated cation channel found in aphids
Source: FEBS Lett. 2015 Feb 27;589(5):598–607. doi: 10.1016/j.febslet.2015.01.020 (PMC4332693; doi:10.1016/j.febslet.2015.01.020)
Supplement: Supplementary data 1 [file mmc9.doc]

**Supplementary data**

**Supplementary Materials and Methods**

**Homology modelling:** A homology model of the *D. melanogaster* DmNa_v_1 selectivity filter was generated using the crystal structure of the bacterial sodium channel NavAb as template (PDB code 3RVY)^30^. Sequences were aligned using ClustalW^52^ and the alignments adjusted according to the model of Tikhonov and Zhorov^53^. 10 models were selected out of 50 produced in MODELLER9v10^46^, based on their objective function score, and the best model was selected after validation of model stereochemistry using the VADAR webserver^54^ ([http://vadar.wishartlab.com](http://vadar.wishartlab.com/)) and visual inspection confirming that side chains of putative TTX-binding determinants faced the lumen.

Using the crystal structure of TTX^48^ (Cambridge Structural Database reference TETXHB), automated docking predictions of TTX in a 12 Å radius site centrally positioned in the selectivity filter of the DmNa_v_1 model were generated using GOLD Version 5.1 [CCDC, Cambridge, UK]. Ten genetic algorithm runs were performed using the following parameters: population size=100; selection pressure=1.1; number of islands=5; niche size=2; migration=10; mutate=95; crossover=95. Side chains projecting into the filter lumen (D377, F378, E985, K1497, Q1501, T1788, S1789 and D1793, according to housefly numbering) were allowed to flex during the docking runs. Docking predictions were evaluated using the GoldScore fitness function^55^.

The top-scoring result (including docked TTX and final rotamer for each flexible side chain) provided the precursor structure for additional docking runs. DeepView software^47^ was used to modify residues to produce the DEKS, DENA or DENS variants of the *D. melanogaster* selectivity filter; the side chains of these changed amino acids were allowed to flex during subsequent docking runs. In GOLD a scaffold constraint weighting of 5 imposed similar orientations of TTX in each selectivity filter enabling the comparison and analysis of docking results. Figures were produced using PyMOL (DeLano Scientific, San Carlos, CA, U.S.A.).

**Supplementary References**

52. Thompson JD, Higgins DG, Gibson TJ. 1994. CLUSTAL W: improving the sensitivity of progressive multiple sequence alignment through sequence weighting, position-specific gap penalties and weight matrix choice. *Nucleic Acids Res*. **22:** 4673–4680. doi:10.1093/nar/22.22.4673.

53.Tikhonov DB, Zhorov BS. 2012. Architecture and pore block of eukaryotic voltage-gated sodium channels in view of NavAb bacterial sodium channel structure. *Mol. Pharmacol.* **82,** 97-104. doi:10.1124/mol.112.078212.

54.Willard L, Ranjan A, Zhang H, Monzavi H, Boyko RF, Sykes BD, Wishart DS. 2003. VADAR: a web server for quantitative evaluation of protein structure quality. *Nucleic Acids Res..* **31:** 3316 –3319. doi:10.1093/nar/gkg565.

55. Jones G, Willett P, Glen RC, Leach AR, Taylor R. 1997. Development and validation of a genetic algorithm for flexible docking. *J. Mol. Biol.* **267:** 727–748. doi:10.1006/jmbi.1996.0897.

**Supplementary data 1: TTX binding model comparison**

Our Na_v_1 pore model is based on the P-loop region of the bacterial Na^+^ channel Na_v_Ab^29^ and differs somewhat from a previous TTX binding model by Fozzard & Lipkind^30^. TTX docks with the unmodified DEKA channel model with a pore-occluding pose that recapitulates a number of experimental findings, including a putative salt bridge between the TTX guanidinium cation and the side chain of E985 (DII) (housefly numbering)^19^. This interaction was also proposed previously by Fozzard & Lipkind^30^, based on a docking of TTX with a model of the Nav1.4 selectivity filter.

As the earlier Fozzard & Lipkind model was generated based on a different structural template (the KcsA potassium channel), features P-loops modelled as a helix-turn-β-strand instead of a helix-turn-helix motif and has TTX bound with an alternative orientation in the vestibule, it is instructive to compare how ligand interactions differ between the two models:

Firstly, TTX is bound deeper in the selectivity filter of the current model where it is too distal to interact with members of the outer ring of negative charges.

Secondly, the D377 side chain forms a hydrogen bond with the C10 hydroxyl group of TTX instead a salt bridge with the TTX guanidinium as in Fozzard & Lipkind^30^.

Thirdly, whereas the DI aromatic residue of the Fozzard & Lipkind model (equivalent to housefly F378) interacts with the non-polar C4-C5-C7-C8 side of TTX, this surface of the ligand instead forms van der Waals contacts with the hydrophobic acyl portion of the K1497 side chain; a hydrogen bond is additionally present between the K1497 side-chain amine and the TTX C4 hydroxyl group. These observations conform to those of Penzotti *et al*., who suggested that the K1497 extended side chain underpins and stabilizes the TTX-bound state.

Finally, the F378 aromatic side chain of the current model is located adjacent to the TTX guanidinium group and is thus positioned to form a cation-π interaction, which is a bond absent from the Fozzard & Lipkind model, yet represents a key experimentally-validated binding contact^20^.
